# Community-Acquired Resistant Strain of Klebsiella pneumoniae in an Elderly Hispanic Patient in the United States: Causing Klebsiella-Invasive Syndrome With a Primary Liver Abscess and Antibiotic Resistance

**DOI:** 10.7759/cureus.83715

**Published:** 2025-05-08

**Authors:** Imad Majeed, Roarke Swank, Naga Chadalapaka, Kenneth Schott Hannan

**Affiliations:** 1 Infectious Diseases, University of Louisville Hospital, Louisville, USA; 2 Internal Medicine, University of Louisville Hospital, Louisville, USA; 3 Internal Medicine, University of Louisville, Louisville, USA

**Keywords:** antibiotic resistance, bacterial liver abscess, extended spectrum beta-lactamase (esbl), gram-negative bacteremia, klebsiella pneumoniae invasive syndrome

## Abstract

Klebsiella pneumoniae (K. pneumoniae) commonly causes respiratory and urinary tract infections but can also lead to community-acquired liver abscesses, primarily in Asian populations. This condition is rare in the Americas and Europe. The emergence of sulfhydryl reagent variable extended-spectrum beta-lactamase (SHV ESBL) strains complicates the management of these abscesses. A Hispanic female in her late 60s presented with bloody urine after three weeks of foul odor, dry cough, headache, and blurry vision. A CT scan of the abdomen and pelvis showed a 1 cm right-sided ureteral calculus and a 5 cm focal liver density, later confirmed as a heterogeneous loculated abscess. A nephroureteral stent was placed. Ophthalmology ruled out endophthalmitis. Blood cultures showed K. pneumoniae, while urine cultures were negative. Cefepime was started based on sensitivity patterns. A CT-guided drain confirmed K. pneumoniae. Quantitative polymerase chain reaction (qPCR) and next-generation sequencing (NGS) revealed a positive SHV ESBL trait. Ertapenem was initiated due to the inability to specify the subtype. Persistent poor drainage prompted a drain change. Repeat imaging showed the reduced abscess size. The patient was discharged on six weeks of intravenous ertapenem with weekly lab tests. This case emphasizes the importance of vigilance for community-acquired Klebsiella liver abscesses in patients with K. pneumoniae infections. Managing liver abscesses, especially those caused by SHV ESBL strains, is challenging due to the lack of specific diagnostic tools. Broad-spectrum antibiotics such as carbapenems are cautiously used to prevent community resistance. Advancements in SHV ESBL studies are crucial for targeted therapy and appropriate antibiotic use.

## Introduction

Klebsiella pneumoniae is a common pathogen that is known to cause liver abscess formation, classically described in Asian countries [1-4]; although rare, emerging cases in Europe and the Americas are increasingly being recognized and described [5-10]. Syndromes and clinical presentations vary; pyogenic liver abscess with bacteremia has been most frequently observed in Asian countries. For example, in Taiwan, one retrospective study found 78% of pyogenic liver abscesses were due to primary K. pneumoniae infection [3], whereas, in another study conducted in New York, USA, around 41% of cases were secondary to K. pneumoniae, with most of these cases occurring in Asian individuals [10]. Typical treatment includes percutaneous drainage with a second- or third-generation cephalosporin; however, pyogenic liver abscess syndrome due to K. pneumoniae still has an overarching mortality of 10% and a 15% rate of metastatic or invasive abscess syndrome [3].

We present a fascinating case of a Hispanic female in her late 60s with primary pyogenic liver abscess syndrome secondary to K. pneumoniae with concurrent bacteremia complicated by a sulfhydryl reagent variable extended-spectrum beta-lactamase pattern resistance. Due to initial concern for primary invasive community-acquired liver syndrome, which has a shockingly high mortality rate, workup discovered a unique resistance pattern in this case that is, to our knowledge, the first described occurrence of its kind, highlighting the need for the development of additional precise sensitivity panels and optimization of antibiotic therapy.

This case was presented at the Annual American College of Physicians Conference in September 2024 in Lexington.

## Case presentation

**Figure 1:**
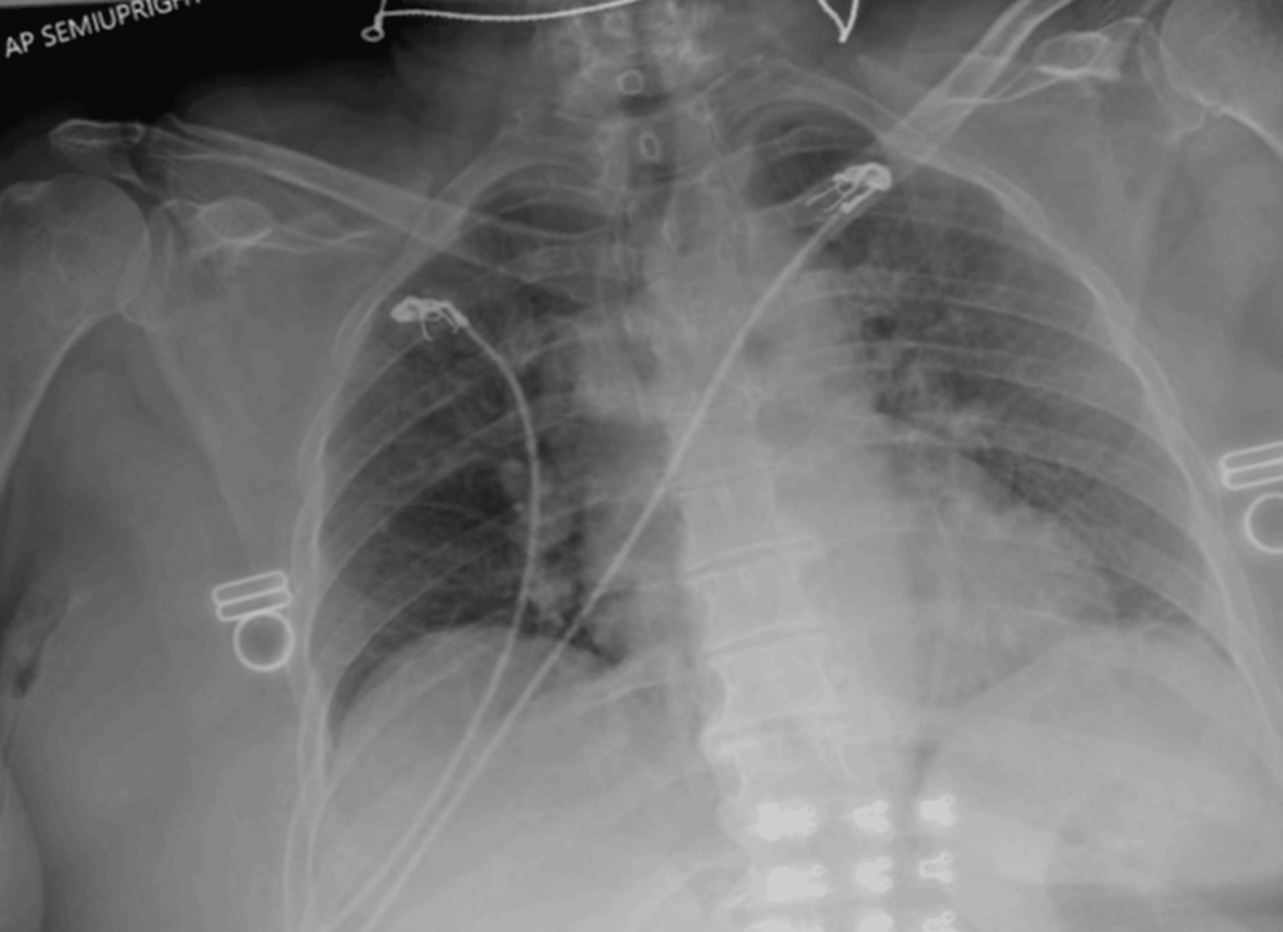
Anterior-posterior chest X-ray revealing mild diffuse interstitial prominence throughout the left greater than the right lung, potentially indicating atypical pneumonia.

A Hispanic female in her late 60s presented to the emergency department with hematuria, foul-smelling urine, headaches, a dry cough, and blurry vision for three weeks. The foul-smelling urine started three weeks from presentation, with headaches, dry cough, blurry vision, and hematuria developing one week from presentation. She also reported polyarthralgia. Past medical history was significant for pre-diabetic status; cholecystectomy was performed nine years prior to presentation. Family and social histories were unremarkable. She did endorse an allergy to penicillin that precipitates a rash. The physical exam demonstrated joint tenderness in the left knee to palpation, otherwise negative; CRP was 328.5 mg/L and ESR 125 mm/hr, which were significantly elevated. The patient was meeting systemic inflammatory response syndrome (SIRS) criteria for sepsis, prompting an infectious disease workup; urinalysis was negative other than large hematuria, and a chest X-ray suggested a mild case of atypical pneumonia (Figure 1).

**Figure 2:**
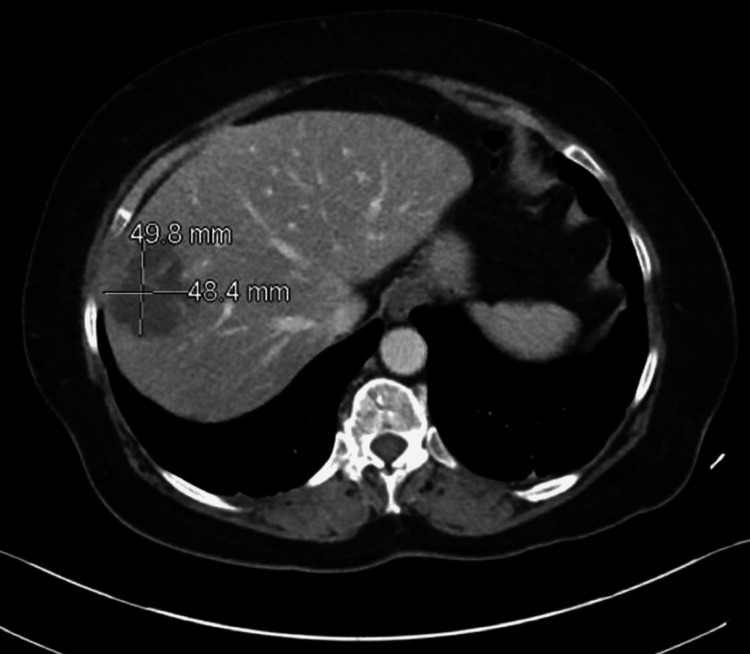
Pre-procedural computed tomography (CT) abdomen demonstrated a 5 cm focal abnormality in the liver parenchyma, consistent with a liver abscess.

A CT without contrast demonstrated a 1-cm obstructing radiolucent stone in the proximal right ureter; urology was consulted. Incidentally, a 5-cm hepatic lesion was detected (Figure 2).

Her bilirubin was elevated (2.1 mg/dL), and with a hepatocellular pattern of liver injury (AST: 76 U/L, ALT: 107 IU/L), an MRI with and without contrast was ordered. She was started on an empiric antibiotic regimen that included cefepime (IV 2 g q8h) and azithromycin (500 mg q12h). Meanwhile, she was tested for other close differentials, causing liver abscesses, with serologic tests for Strongyloides and Entamoeba histolytica antibodies, along with stool for ova and parasites, which came back negative later in the hospital course.

**Figure 3:**
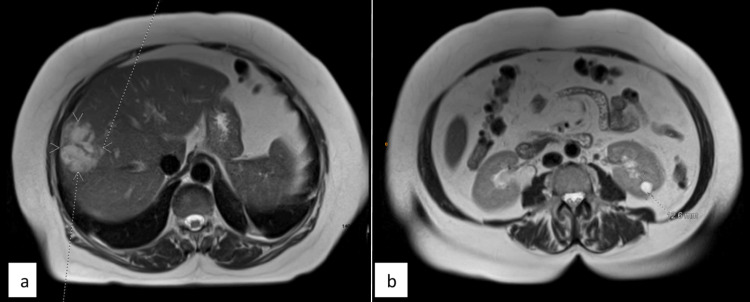
(a) An axial view of the T2-weighted image of the MRI abdomen and pelvis demonstrates a multiloculated right hepatic lobe lesion accompanied by surrounding reactive hyperemia, suggesting a liver abscess. (b) An axial view of the T2-weighted image of the MRI abdomen and pelvis reveals an obstructing calculus in the right mid ureter, leading to moderate upstream hydroureteronephrosis. In Figure 3a, the dotted arrowheads in white color indicate the multiloculated right hepatic abscess, while the dotted lines in the background are artifacts.

On day two, the MRI identified the hepatic lesion as a 5-cm heterogeneous, loculated abscess (Figures 3a-3b).

**Figure 4:**
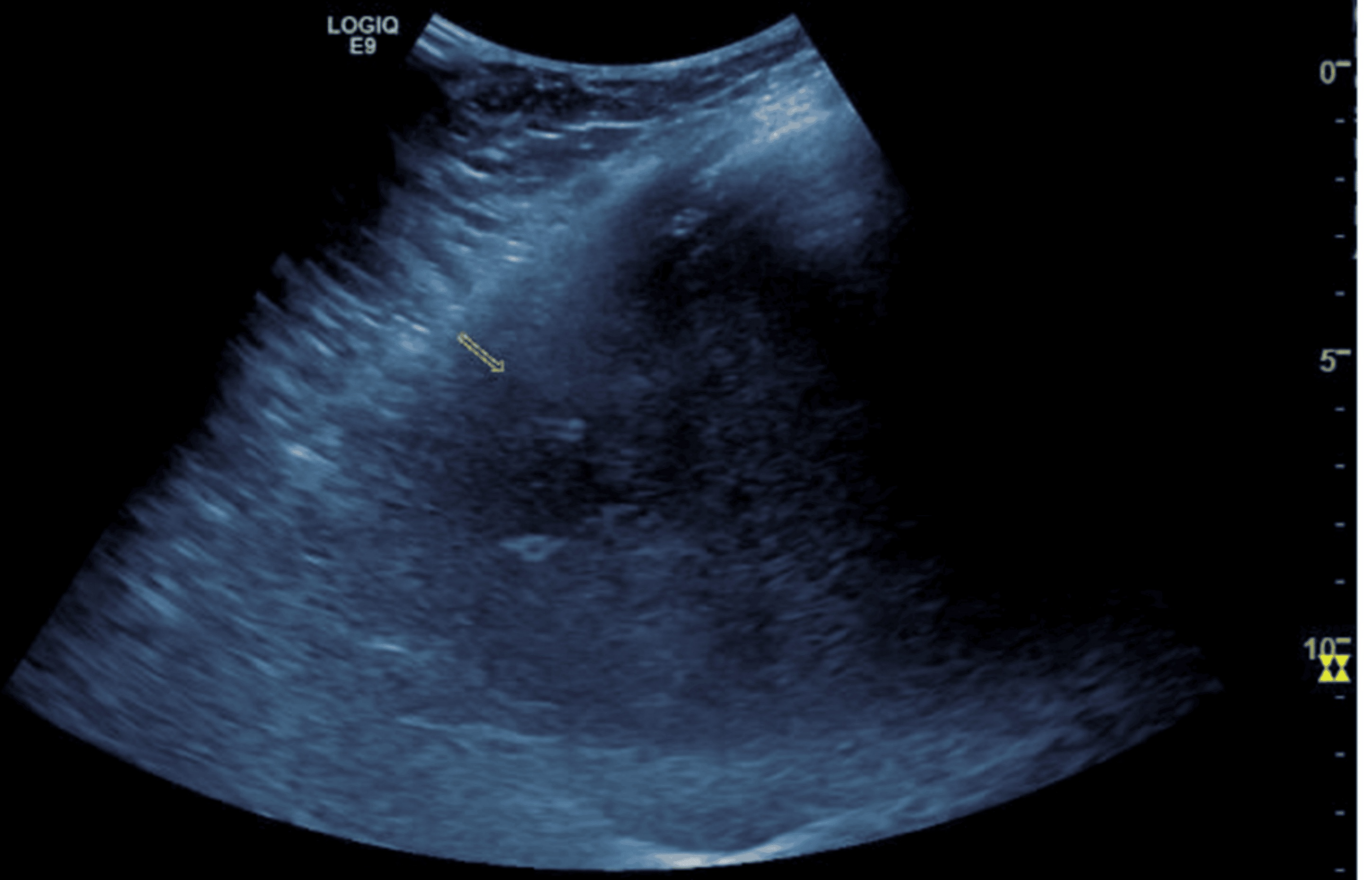
This ultrasound image depicts the right upper quadrant of the abdomen, revealing a hypoechoic abscess in the right hepatic lobe. The abscess measures approximately 4.4 cm x 3.1 cm x 4.0 cm and contains a partially imaged pigtail drainage catheter. The central components of the abscess exhibit liquefaction. The yellow small arrow indicates the right hypoechoic abscess.

At the same time, blood cultures obtained on the day of admission were positive for K. pneumoniae, with sensitivities pending initially. Based on new-onset blurry vision, headaches, Klebsiella bacteremia, polyarthralgia, and a hepatic abscess, clinical concern for community-acquired K. pneumoniae invasive syndrome complicated by a primary liver abscess was established. Interventional radiology was consulted for ultrasound-guided drain placement and fluid culture collection (Figure 4); infectious disease recommended adding Microgen (next-generation sequencing and quantitative polymerase chain reaction) to precisely evaluate the etiology of the hepatic abscess, ophthalmology was consulted to rule out endophthalmitis, and a transesophageal echocardiogram was ordered to rule out infective endocarditis. Metronidazole (IV 500 mg q8 h) was initiated, and azithromycin was discontinued due to low concern for atypical pneumonia (Legionella and streptococcal pneumonia urine antigen were negative). Urology placed a ureteral stent to alleviate obstructive hydronephrosis; repeated urinalysis was also negative for urinary tract infection.

**Figure 5:**
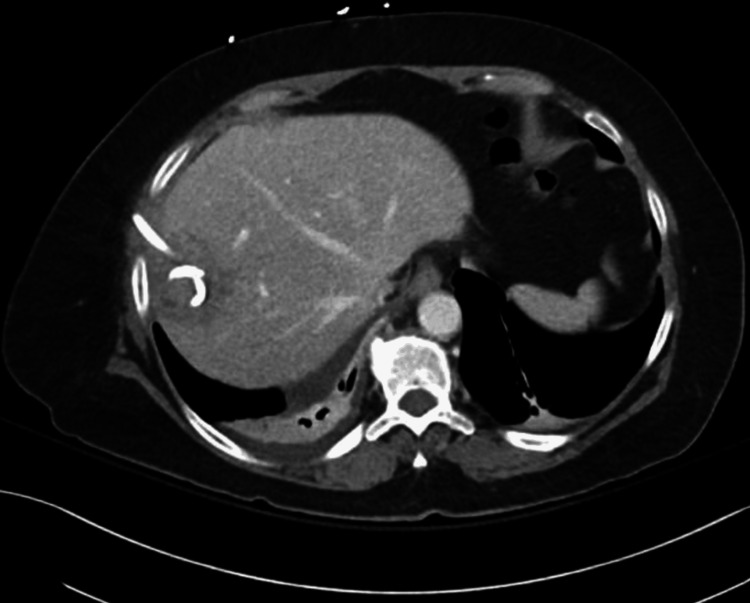
Post-procedural CT abdomen revealed the placement of a surgical drain within the liver abscess and its drainage. The white opaque object seen in the right lobe of the liver at the lesion site suggests the surgical drain.

Then, ophthalmology ruled out endophthalmitis as the cause of the blurry vision, and the transesophageal echocardiogram was negative for vegetative disease, although moderate tricuspid regurgitation was identified. On day five, initial sensitivity culture results indicated extended-spectrum beta-lactamase positivity, prompting a switch to IV cefepime 2 g every eight hours. Repeat blood cultures were negative at day five. On day seven, repeat CT demonstrated persistent abscess presence with no change in size (Figure 5).

**Figure 6:**
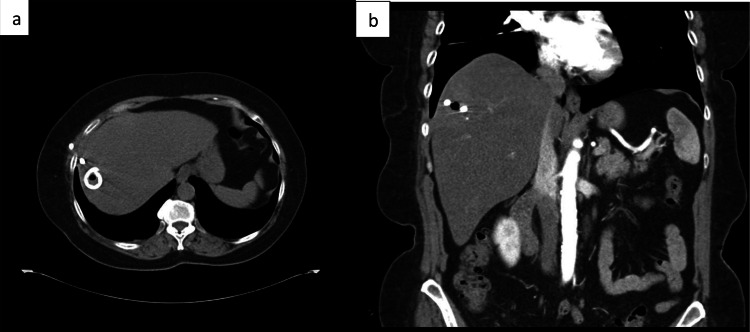
(a) Transverse section of the computed tomography (CT) abdomen and pelvis obtained at a follow-up visit is presented. This image demonstrates the presence of a Pigtail catheter in the right liver, with a small focal area of air within the hepatic parenchyma. No discernible residual drainable collection is identified at this time. (b) Sagittal section of the CT abdomen and pelvis, with consistent findings. The white opaque object in the right lobe of the liver at the previous lesion site, suggests the Pigtail catheter with residual air and resolved a prior liver abscess.

This prompted a drain exchange on day nine for a larger gauge to encourage abscess drainage. At this time, the Microgen test result was finalized, further indicating an unexpected finding of sulfhydryl reagent variable extended-spectrum beta-lactamase-positive K. pneumoniae. Finally, by day 10, metronidazole and cefepime were discontinued, and monotherapy with IV ertapenem at 1 g q24h was initiated; six weeks of monotherapy with a midline placement on an outpatient basis was scheduled, with a two-week follow-up appointment, to remove the hepatic drain. At that time, repeat CT of the abdomen and pelvis revealed complete resolution of the abscess with no evidence of residual infection (Figures 6a-3b), and the patient made a full recovery.

## Discussion

K. pneumoniae is a Gram-negative, non-motile bacterium that frequently colonizes human mucosal surfaces, such as those in the gastrointestinal tract and oropharynx [11]. It can spread from these colonizing sites to other sterile tissues, causing infections such as urinary tract infections, pneumonia, and others in humans [12]. Reports have been published on the emergence and dissemination of hypervirulent (HV) or antibiotic-resistant strains of K. pneumoniae [13]. The factors most associated with its virulence include lipopolysaccharides, capsules, siderophores, and fimbriae (known as pili) [12]. Among these, the capsular component, synthesized by genes from the capsular polysaccharide synthesis (cps) locus, has been extensively studied for its role in virulence [11]. Seventy different capsular serotypes have been identified in the literature, with K1 and K2 serotypes most frequently associated with liver abscesses [14,15].

In our patient, a mucoid strain of K. pneumoniae was detected in both blood culture and abscess aspirate, though it was not further classified into subtypes. K. pneumoniae has emerged as a significant cause of liver abscesses in the USA, with several reports published over the years. A review article identified 96 case reports in the USA, primarily involving patients of Asian descent. Only a few cases were reported in patients of Hispanic or Caucasian ethnicity, suggesting a possible genetic predisposition among individuals of Asian descent, which may explain the higher prevalence of Klebsiella-associated liver abscesses in this population [15]. The mechanism by which Klebsiella causes primary liver abscesses is not completely understood, but there is an observed association with diabetes and pre-diabetic conditions. Impaired glucose tolerance may contribute to the spread of infection by reducing neutrophil function, which is critical for breaking down the bacterial capsule [15].

Our patient had an HbA1c of 6.1 mmol/mol, placing her in the pre-diabetic range and increasing her risk of developing a primary liver abscess and bacteremia secondary to K. pneumoniae. K. pneumoniae is capable of infecting healthy individuals, particularly through its HV strains, and can lead to community-acquired infections, such as pyogenic liver abscesses, meningitis, necrotizing fasciitis, endophthalmitis, and severe pneumonia [13].

Liver abscesses can be caused by bacterial, parasitic, or fungal pathogens. These pathogens can infect the liver through various routes, including the portal vein, biliary tree, hepatic artery, direct extension, or penetrating trauma [15]. In our case, the patient’s history of ureteral stones and diverticulosis likely contributed to the development of K. pneumoniae bacteremia and subsequent primary liver abscess. Most K. pneumoniae hospital-acquired infections originate from gastrointestinal tract colonization, which may extend to the urinary tract, respiratory tract, or bloodstream [16]. Klebsiella can also form biofilms on medical equipment (e.g., catheters and endotracheal tubes), leading to infections [17].

A review of the literature indicates that patients with K. pneumoniae-associated primary liver abscesses are typically treated with a combination of IV antibiotics and percutaneous liver drainage. Common antibiotic regimens include IV ceftriaxone and metronidazole, piperacillin-tazobactam, ceftriaxone alone, gatifloxacin, imipenem/levofloxacin, cefepime, ciprofloxacin/imipenem, or penicillin/gentamicin/metronidazole [15]. Our patient was initially treated with IV cefepime and metronidazole, along with percutaneous abscess drainage. However, due to the diverse resistance pattern of K. pneumoniae, it has developed resistance to beta-lactam antibiotics [12].

A specimen from the abscess aspirate was sent for Microgen testing (next-generation sequencing), which revealed the presence of a resistant SHV beta-lactamase gene. The report also indicated resistance to fourth-generation cephalosporins. A timely decision was made to switch the patient’s antibiotic therapy to IV ertapenem, which prevented further complications. The SHV beta-lactamase gene confers resistance to various beta-lactam antibiotics. According to a review article, SHV genes are categorized into subgroups 2b, 2br, and 2be. Subgroup 2b is resistant to penicillin and early cephalosporins, subgroup 2br is resistant to clavulanic acid, and subgroup 2be is resistant to third- and fourth-generation cephalosporins [18].

We attempted to contact the Microgen (NGS and qPCR) testing facility to further classify the SHV-resistant gene into its specific subgroup. However, this was not performed, possibly due to unavailable testing methods. This report highlights the need for further subclassification of SHV beta-lactamase-resistant genes in K. pneumoniae. Early detection could help prevent complications and improve patient outcomes.

## Conclusions

This case underscores the significance of vigilance in detecting community-acquired Klebsiella liver abscesses among patients with K. pneumoniae infections. Managing liver abscesses, particularly those caused by SHV ESBL strains, presents significant challenges due to the absence of specific diagnostic tools. Consequently, broad-spectrum antibiotics such as carbapenems are cautiously employed to prevent the development of community resistance. Ongoing research on SHV ESBLs is essential for developing targeted therapies and ensuring appropriate antibiotic usage.
